# MiR-125a-5p decreases after long non-coding RNA *HOTAIR* knockdown to promote cancer cell apoptosis by releasing caspase 2

**DOI:** 10.1038/cddis.2016.41

**Published:** 2016-03-10

**Authors:** L Tang, H Shen, X Li, Z Li, Z Liu, J Xu, S Ma, X Zhao, X Bai, M Li, Q Wang, J Ji

**Affiliations:** 1 State Key Laboratory of Protein and Plant Gene Research, College of Life Sciences, Peking University, Beijing, China; 2 Key Laboratory of Genomics and Precision Medicine, China Gastrointestinal Cancer Research Center, Beijing Institute of Genomics, Chinese Academy of Sciences, Beijing, China; 3 Institute of Forensic Science, Ministry of Public Security, Beijing, China; 4 Department of General Surgery, Beijing ChaoYang Hospital, Capital University of Medical Sciences, Beijing, China

## Abstract

*HOTAIR* (homeobox transcript antisense RNA), one of the prototypical long non-coding RNAs, has been verified overexpressed in multiple carcinomas and has emerged as a promising novel anticancer target. Its well-established role is acting as a predictor of poor prognosis and promoting cancer cell metastasis. Recently, another important mission of *HOTAIR* was uncovered that targeting *HOTAIR* caused cancer cell apoptosis. Nevertheless, so far there is no published data elaborating the mechanism. Here, we report that microRNA miR-125a-5p decreases and releases caspase 2 to promote cancer cell apoptosis after *HOTAIR* knockdown. We applied siRNAs targeting *HOTAIR* to various cancer cells, and observed apoptosis in all of these cell lines. RNA sequencing detected that miR-125a-5p was decreased after *HOTAIR* knockdown and miR-125a-5p mimics could rescue the apoptosis induced by *HOTAIR* deficiency. Luciferase assays identified caspase 2, an initiator caspase, to be a new target of miR-125a-5p. Elevated expression and subsequent cleavage of caspase 2 was observed after *HOTAIR* knockdown or inhibition of miR-125a-5p. RNAi of caspase 2 could attenuate the apoptosis induced by *HOTAIR* knockdown. In 80 clinical colon cancer tissues, *HOTAIR* and miR-125a-5p levels were higher than adjacent tissues, whereas caspase 2 was lower. MiR-125a-5p expression level was significantly correlated with colon tumor size, lymph node metastasis and clinical stage. These findings indicate that miR-125a-5p decreases after *HOTAIR* knockdown to promote cancer cell apoptosis by releasing caspase 2. Our work reveals a previously unidentified apoptotic mechanism, which might be exploitable in anticancer drug development.

*HOTAIR* (homeobox transcript antisense RNA), the first discovered trans-acting long non-coding RNA (lncRNA), has been validated having an unignorable role in oncogenic progression. It was discovered aberrantly overexpressed in many kinds of cancer, including acute myeloid leukemia,^1^ cervical cancer,^2^ liver cancer,^3^ urothelial cancer,^4^ oral squamous cell carcinoma,^5^ breast cancer,^6^ glioma cancer,^7, 8^ ovarian cancer^9^ and gastric cancer,^10, 11^ suggesting a promising therapeutic target role in broad-spectrum cancer treatment. The high expression level of *HOTAIR* in tumors is a powerful predictor of eventual metastasis and bad prognosis.^10, 11^ The possible metastatic mechanism is that *HOTAIR* interacts with polycomb repressive complex 2 (PRC2) and lysine-specific demethylase 1A (LSD1) to epigenetically alter the expression of *HOXD* and some other select genes.^12, 13^ Although this metastasis-promoting theory is innovative and convincing, it might not fully illustrate *HOTAIR*'s significant role in carcinogenesis.

Recently, a notable mission of *HOTAIR* that it is essential for cancer cell survival has been identified. It was reported that knockdown of *HOTAIR* sensitized HepG2 cells to TNF-*α*, doxorubicin and cisplatin, and the level of *HOTAIR* declined markedly in HeLa and MCF-7 cells when apoptosis induced by bleomycin happened.^12, 13^ Moreover, *HOTAIR* knockdown caused apoptosis in multiple cancer cell lines, and ectopic expression of *HOTAIR* reduced that.^9, 14, 15, 16^ Tumor shrinking effect of targeting *HOTAIR* has been validated in a mouse xenograft model.^16^ However, there is not a study reporting why *HOTAIR* knockdown leads to cancer cell apoptosis. As inducing apoptosis is an universal cancer therapeutic regimen, elucidating how apoptosis is triggered would be illuminating for cancer treatment. In this study, we discovered that microRNA miR-125a-5p decreased after *HOTAIR* knockdown, and its decline derepressed translation of its target, caspase 2(*Casp2*), and thus caused self-cleavage of CASP2 and activation of the mitochondrial apoptosis pathway. This newly uncovered apoptotic mechanism might be valuable in cancer therapy.

## Results

### *HOTAIR* knockdown leads to cancer cell apoptosis

**Figure 1:**
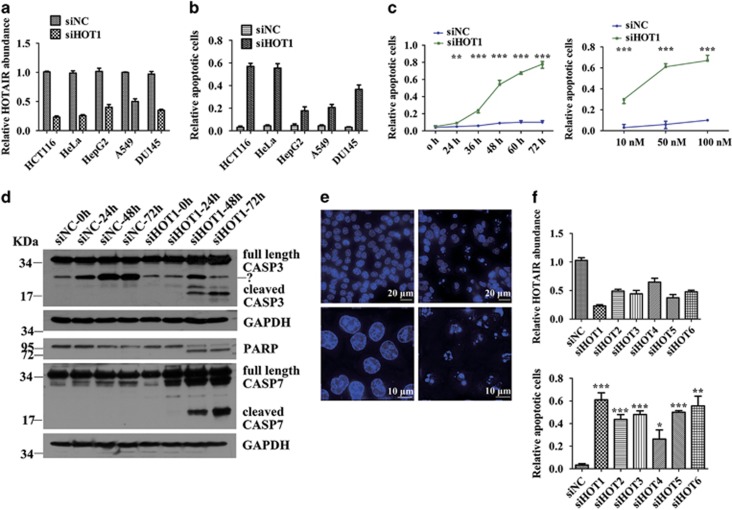
*HOTAIR* knockdown leads to cancer cell apoptosis. (**a**) Quantitative real-time PCR measured the relative abundance of *HOTAIR*. (**b**) FCM detected the apoptotic situation of cells transfected with 50 nM siHOT1 or siNC for 48 h. (**c**) Apoptosis happened in a siHOT dose- and time-dependent manner in HCT116 cells. (**d**) Western blot detected the cleavage of CASP3, CASP7 and PARP. (**e**) Hoechst staining showed nuclear condensation of HCT116 cells. (**f**) Upper, quantitative real-time PCR measured the relative abundance of *HOTAIR* after 24-h transfection of 50 nM siHOT1-6 or siNC. Lower, FCM detected the apoptotic situation of cells transfected with 50 nM siHOT1-6 or siNC for 48 h. Bars represent mean±S.E.M. from three independent experiments. *P<0.05, **P<0.01 and ***P<0.001 by Student's *t*-test. All experiments were performed in three biological repeats

In order to find out whether *HOTAIR* is essential for cancer cell survival, we transfected a specific siRNA targeting *HOTAIR* (siHOT1) or scrambled siRNA (siNC) into HCT116, HeLa, HepG2, A549 and DU145 cells. Apoptotic assays were carried out after 48-h transfection. Obvious apoptosis was detected by flow cytometry (FCM) in all of these five cell lines. The amount of apoptotic cells was about 60% in HCT116 and HeLa cells, 20% in HepG2 and A549 cells, and 40% in DU145 cells (Figure 1b). Quantitative real-time PCR confirmed the effective knockdown of *HOTAIR* in all of these cell lines (Figure 1a). The amount of apoptotic cells significantly increased in a *HOTAIR* RNAi dose and time-dependent manner (Figure 1c, Supplementary Figures S1a and S1b). Caspase 3 (CASP3), Caspase 7 (CASP7) and poly ADP-ribose polymerase (PARP) cleavage was detected by western blot in HCT116 cells transfected with siHOT1 (Figure 1d). Morphology change in the compaction of nuclear chromatin is one of the characteristics of apoptotic execution. A remarkable degree of DNA compaction was observed in HCT116 cells transfected with siHOT1 (Figure 1e). To ensure these results were not off-target effects of RNA interference, we used six independent siRNA sequences targeting different sites of *HOTAIR*. Each siRNA depleted *HOTAIR* and led to concomitant apoptosis (Figure 1f, Supplementary Figure S1c). These data suggest that *HOTAIR* is vital for cancer cell survival and deficiency of *HOTAIR* leads to cancer cell apoptosis.

### MiR-125a-5p declines after *HOTAIR* knockdown and inhibits apoptosis

**Figure 2:**
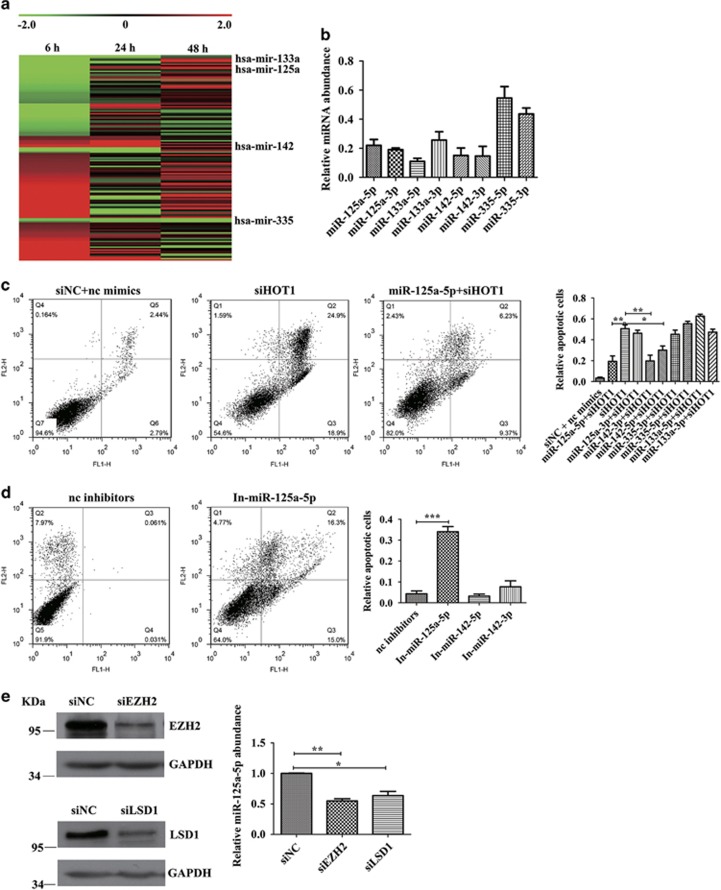
MiR-125a-5p is reduced after *HOTAIR* knockdown. (**a**) Heat map of genes with significant induction (red) or repression (green) at sixth hour after 50 nM siHOT1 transfection into HCT116 cells (expressed as a ratio to HCT116 cells transfected with siNC, fold change >2, *P*-value <0.05, data are log_2_ transformed). (**b**) Quantitative real-time PCR validation of a representative panel of genes detected down-regulation by RNA-seq. Raw Ct values were normalized to U6 RNA. Bars represent mean±S.E.M. from three independent experiments. (**c**) FCM detected the rescue effect of miRNAs on apoptosis induced by *HOTAIR* knockdown. (**d**) Inhibition of miR-125a-5p caused apoptosis. (**e**) miR-125a-5p declined after RNAi of EZH2 and LSD1. Bars represent the mean±S.E.M. from three independent experiments. *P<0.05, **P<0.01 and ***P<0.001 by Student's *t*-test. All experiments were performed in three biological repeats

To figure out why *HOTAIR* is vital for cancer cell survival, we performed RNA deep sequencing to screen ncRNAs regulated by *HOTAIR*. Total RNA of cells transfected with siHOT1 for 6, 24 and 48 h was extracted and sequenced. Among the 8025 identified long ncRNAs, most are unannotated new lncRNAs and some are pri-miRNAs. Based on fold change >2 and *P-*value <0.05, 145 long ncRNAs were dysregulated at the sixth hour after siHOT1 transfection, among which the expression of pri-miRNAs hsa-mir-125a, hsa-mir-133a, hsa-mir-142 and hsa-mir-335 declined markedly (Figure 2a). The decline of these pri-miRNAs' processed mature miRNAs was validated with quantitative real-time PCR (Figure 2b). Considering that pri-miRNAs are processed to yield mature 22-nucleotide miRNAs to execute their function, we used miRNA mimics and inhibitors to explore the function of these miRNAs. To restore these miRNAs' expression level, we transfected miRNA mimics into HCT116 cells 6 h before the transfection of siHOT1. MiR-125a-3p, miR-133a-5p, miR-133a-3p, miR-335-5p and miR-335-3p did not rescue the apoptosis caused by *HOTAIR* knockdown, whereas miR-142-5p, miR-142-3p and miR-125a-5p markedly suppressed the apoptosis (Figure 2c). These data indicate that miR-142-5p, miR-142-3p and miR-125a-5p can inhibit apoptosis. We transfected inhibitors of these three miRNAs into HCT116 cells and observed that reduction of miR-125a-5p triggered apoptosis, whereas miR-142-5p and miR-142-3p did not (Figure 2d). Although miR-125a-5p and miR-125a-3p were both downregulated, only miR-125a-5p could reduce the apoptosis induced by *HOTAIR* deficiency. Inhibition of miR-125a-5p triggered apoptosis without any additional stimulation. These results suggest that miR-125a-5p, as a suppressor of apoptosis, has a significant role in *HOTAIR* knockdown induced apoptosis.

To find out the mechanism that *HOTAIR* regulates the expression of miR-125a-5p, we silenced the co-factors of *HOTAIR*, enhancer of zeste homolog 2 (EZH2) and LSD1, using siRNAs, and detected the level of miR-125a-5p with quantitative real-time PCR. The level of miR-125a-5p decreased significantly with the suppression of EZH2 and LSD1 (Figure 2e), suggesting that the transcription of miR-125a-5p might be epigenetically regulated by *HOTAIR* and its co-factors EZH2 and LSD1.

### CASP2 is a target of miR-125a-5p and an initiator in *HOTAIR* deficiency-induced apoptosis

**Figure 3:**
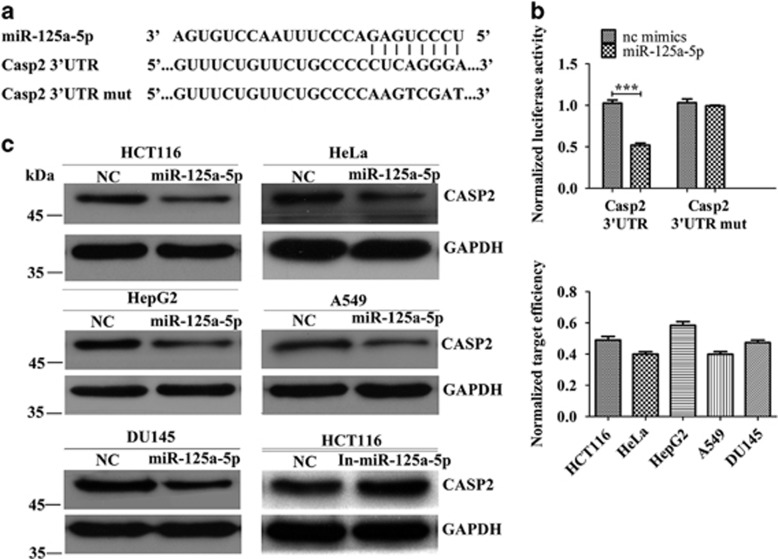
*Casp2* is a target of miR-125a-5p. (**a**) Schematic outlines the predicted binding sites of miR-125a-5p on *Casp2*. (**b**) Luciferase reporter assay confirmed the target effect of miR-125a-5p on *Casp2*. (**c**) Western blot confirmed the target effect of miR-125a-5p on Casp2. Gray value of each CASP2 band was normalized to GAPDH to calculate target efficiency. Bars represent the mean±S.E.M. from three independent experiments. ***P<0.001 by Student's *t* -test. All experiments were performed in three biological repeats

Given that miRNAs function by targeting mRNAs, we used *in silico* miRNA target prediction tools PicTar and TargetScan to predict potential target mRNAs of miR-125a-5p. *Casp2* mRNA was predicted as high-confidence miR-125a-5p target. In all, 200-bp fragments containing wild-type or random mutated putative miR-125a-5p binding sites of *Casp2* mRNA' 3′UTR were inserted into psiCHECK™-2 vector (Figure 3a). MiR-125a-5p reduced the luciferase activities of psiCHECK™-2-CASP2 by 50%, whereas the luciferase activities of psiCHECK™-2-CASP2-mut did not (Figure 3b). These data suggest that miR-125a-5p can bind to and repress the translation of *Casp2* mRNA. To demonstrate that, we transfected 50 nM miR-125a-5p mimics or 100 nM miR-125a-5p inhibitors (In-miR-125a-5p) into HCT116, HeLa, HepG2, A549 and DU145 cells. Western blot detected about 50% decrease in CASP2 by miR-125a-5p mimics and onefold increase by miR-125a-5p inhibitors (Figure 3c). These results show that *Casp2* mRNA is a target of miR-125a-5p.

**Figure 4:**
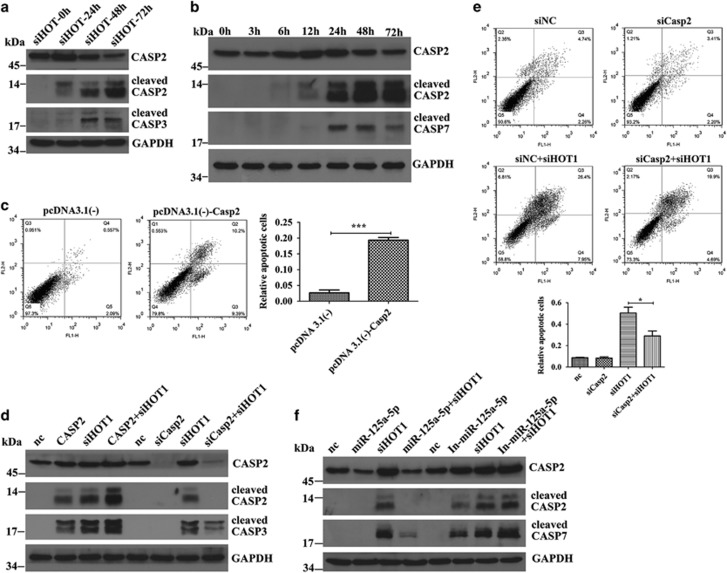
CASP2 serves as an initiator in *HOTAIR* knockdown induced apoptosis. (**a**) Upregulation of full-length CASP2 and cleavage of CASP2 and CASP3 was detected after HOTAIR knockdown. (**b**) Overexpressed CASP2 cleaved itself and activated CASP7. (**c**) Overexpression of CASP2 induced apoptosis in HCT116 cells. (**d**) pcDNA3.1(-)-CASP2 aggravated activation of CASP3 and siCasp2 reduced that. (**e**) SiCasp2 rescued the apoptosis caused by siHOT1. (**f**) MiR-125a-5p diminished cleavage of CASP2 and CASP7 and inhibition of miR-125a-5p aggravated that. Bars represent the mean±S.E.M. from three independent experiments. *P<0.05, ***P<0.001 by Student's *t*-test. All experiments were performed in three biological repeats

As miR-125a-5p declined after *HOTAIR* knockdown and miR-125a-5p targets *Casp2*, we checked the level of CASP2. As shown in Figure 4a, the level of full-length CASP2 markedly increased and then decreased after the silencing of *HOTAIR*. Cleavage of CASP2 and CASP3 was also detected. These data demonstrate that accumulation and activation of CASP2 happened after *HOTAIR* RNAi. It has been reported that CASP2 is an initiator protease of the mitochondrial apoptotic pathway and elevated CASP2 can cleave and activate itself to induce apoptosis. To confirm that, we transfected pcDNA3.1(-)-CASP2 into HCT116 cells. After 6 h, western blot detected a slight increase in full-length CASP2 protein but remarkable production of cleaved CASP2. Cleavage of CASP7 also occurred (Figure 4b). FCM detected about 20% early and advanced apoptotic cells about 36 h after pcDNA3.1(-)-CASP2 transfection (Figure 4c). These results prove that piling up of CASP2 contributes to self-activation, caspase cascade and apoptosis. We supposed that the accumulation of CASP2 protein after *HOTAIR* knockdown might be one of the incentives to apoptosis. To test that, we transfected siRNA targeting *Casp2* into HCT116 cells. As shown in Figures 4d and e, the cleavage of CASP3 and the amount of apoptotic cells were diminished, suggesting that CASP2 contributed to apoptosis at upstream of the mitochondrial apoptotic pathway. When miR-125a-5p mimics were co-transfected with siHOT1, the increase in CASP2 was impaired and the cleavage of CASP2 and CASP3 was weakened. Consistently, inhibition of miR-125a-5p gave rise to increase in full-length CASP2 and aggravated cleavage of CASP2 and CASP7 (Figure 4f). These data indicate that CASP2 is modulated by miR-125a-5p to execute its apoptotic initiator role. This part of the results imply that when miR-125a-5p declines after *HOTAIR* knockdown, its target mRNA *Casp2* is released, leading to accumulation of apoptosis initiator CASP2. The accumulated CASP2 cleaves itself and activates mitochondrial apoptosis pathway, finally resulting in apoptosis.

Besides, we detected the level of P53 and B-cell lymphoma 2 (BCL-2) after silencing *HOTAIR* and found that the level of BCL-2 did not change while P53 was upregulated, implying a potential role of P53 in the apoptosis caused by HOTAIR deficiency (Supplementary Figure S2a). In addition, we confirmed that P53 was a target of miR-125a-5p (Supplementary Figures S2b–d). Taken together, after *HOTAIR* was knocked down, because of the declined expression of miR-125a-5p, its apoptotic targets CASP2 and P53 were accumulated and finally lead to apoptosis.

### MiR-125a-5p is an independent marker of colon cancer progression

**Figure 5:**
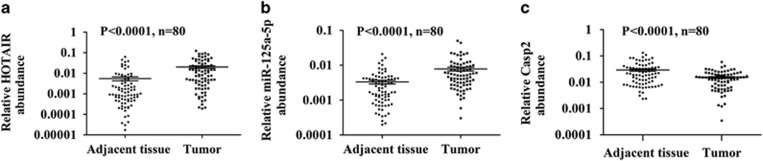
*HOTAIR*, miR-125a-5p and *Casp2* expression in 80 pairs human colon cancer tissues and adjacent noncancerous tissues. (**a**) The dot represents relative *HOTAIR* abundance of each sample. The expression level of *HOTAIR* was calculated by the 2^ΔCt^ method, ΔCt=Ct(GAPDH)—Ct (HOTAIR). The Y axis was log_10_ transformed. (**b**) The expression level of miR-125a-5p was normalized to U6 RNA. (**c**) The expression level of *Casp2* was normalized to GAPDH. The bars illustrated S.E.M. and the significant differences between samples were analyzed using Student's *t*-test

To evaluate the expression level of *HOTAIR,* miR-125a-5p and *Casp2* in colon cancer cells, we performed quantitative real-time PCR in 80 paired cancerous and adjacent noncancerous tissues of colon cancer patients (Table 1). As shown in Figure 5, 63 cases (79%) exhibited higher levels of *HOTAIR* in tumors than in adjacent nontumorous tissues (mean ratio of 3.68-fold, *P*<0.0001). MiR-125a-5p level was significantly elevated in 64 cancerous tissues (80%) (mean ratio of 2.36-fold, *P*<0.0001), whereas *Casp2* was decreased in 59 tumorous tissues (74%) (mean ratio of 0.52-fold, *P*<0.0001). Higher *HOTAIR* level has been proved to be associated with larger tumor size, advanced pathological stage, extensive metastasis and poorer survival. Based on the Mann–Whitney test, the high miR-125a-5p group showed greater incidence of bigger tumor size (*P*=0.023), lymph node metastasis (*P*=0.037) and clinical stage (*P*=0.025). No significant correlation between miR-125a-5p level and patient age (*P*=0.981) or gender (*P*=0.992) was found (Table 1). These results suggest that miR-125a-5p is an independent marker of colorectal cancer progression.

## Discussion

*HOTAIR*, a typical trans-acting lncRNA, is highly expressed in a variety of cancers and thus have emerged as a potential anticancer target. Its well-established role is that the increased *HOTAIR* expression is a biomarker of poor prognosis, and it acts as a modular scaffold of histone modification complex PRC2 and LSD1 to regulate the expression of select genes and promote cancer cell metastasis.^12, 17, 18^ Recently, another significant role of *HOTAIR* was reported and that targeting *HOTAIR* led to cancer cell apoptosis. However, the mechanism remained unknown. In this study, we unraveled that miR-125a-5p decreased after *HOTAIR* knockdown, which brought about cleavage of proapoptosis protein CASP2, and therefore activated the mitochondrial apoptosis pathway.

Yang Z *et al.*^12^ reported that RNAi of *HOTAIR* sensitized HepG2 cells to TNF-*α*, doxorubicin and cisplatin. The expression of *HOTAIR* decreased in cells treated with calycosin, genistein or bleomycin.^13, 17^ *HOTAIR* knockdown induced apoptosis in multiple kinds of cancer cells, and overexpression of *HOTAIR* inhibited it.^8, 9, 14, 15, 16^ In our study, we observed apoptosis in all the cell lines transfected with siHOT and found a dose- and time-dependent correlation between cell viability and siHOT. Remarkable DNA compaction and pronounced CASP3, CASP7 and PARP cleavage took place after *HOTAIR* knockdown. All of these results confirmed the previous notion that *HOTAIR* deficiency led to cancer cell apoptosis. However, we did not detect any remedial effect of *HOTAIR* on apoptosis induced by cisplatin or TNF-*α* (data not shown), which was inconsistent with previous research.^15^ This inconsistency may be due to different apoptosis inducers. In summary, except for the role of promoting metastasis and predicting bad prognosis, *HOTAIR* is vital for cancer cell survival and its knockdown causes cancer cell apoptosis; therefore targeting *HOTAIR* might be promising in cancer therapy.

MiR-125 family is composed of three homologs, miR-125a, miR-125b-1 and miR-125-2. MiR-125a gene is located at 19q13 and processing of hsa-mir-125a generates miR-125a-5p and miR-125a-3p. They target different mRNAs and own controversial properties in different cellular context. MiR-125a-3p was reported suppressing proliferation and migration and inducing apoptosis in multiple cancer cells.^18, 19^ Unlike miR-125a-3p, miR-125a-5p acts as either oncogene or suppressor gene depending on the specific cell context. MiR-125a-5p is downregulated in breast cancer,^20^ ovarian cancer^21^ and lung cancer.^22^ It inhibits glioblastoma cell proliferation by targeting tafazzin.^23^ On the other hand, higher expression level of miR-125a-5p is observed in nasal pharyngeal cancer cells, multiple myeloma cells, human prostate cancer cells and the serum of non-small-cell lung cancer patients.^24, 25, 26, 27^ By targeting P53 directly, it promotes proliferation, migration and invasion of HONE1 cells.^24^ Inhibition of miR-125a-5p in multiple myeloma cells reduced cell growth, increased apoptosis and dampened cell migration.^25^ In our study, we observed that the level of pri-miRNA hsa-mir-125a and its mature forms miR-125a-5p and miR-125a-3p declined after inhibition of *HOTAIR*, and miR-125a-5p declined after inhibition of EZH2 and LSD1, which imply that the transcription of hsa-mir-125a might be managed by *HOTAIR* and its epigenetic co-factors EZH2 and LSD1. Although PRC2 complex was usually considered to suppress gene expression, research has proven that it can also promote gene expression.^28, 29^ Unlike miR-125a-3p, miR-125a-5p markedly reduced the apoptosis caused by *HOTAIR* knockdown and inhibition of miR-125a-5p led to apoptosis in HCT116 cells. We also observed that miR-125a-5p functions as an oncogene in colon cancer cells via targeting *Casp2* and *P53*. Furthermore, just like *HOTAIR*, the amount of miR-125a-5p in colon cancerous tissues was more than that in adjacent healthy tissues, and it was positively correlated with tumor size, lymph node metastasis and clinical stage. These results reveal that miR-125a-5p is an oncogene in colon cancer cells and it is an independent marker for colon cancer. Since miR-125a-5p efficiently rescued the apoptosis triggered by *HOTAIR* knockdown, it's possible that miR-125a-5p acts at upstream of the apoptosis signaling pathway.

As CASP2 displays the properties of both initiator and effector caspase, it is hard to define CASP2 as a canonical initiator or effector. Like the initiator caspase 9 (CASP9), CASP2 contains a N-terminal caspase recruitment domain (CARD), followed by a large subunit containing the active site (p19) and a small subunit (p12).^30^ Its activation occurs by proximity-induced dimerization and autoproteolysis.^31^ Ectopic overexpression of CASP2 is sufficient for its activation.^32, 33, 34^ Activated CASP2 arouses caspase cascade and apoptosis via cleaving BH3 interacting domain death agonist (BID) and releasing cytochrome c into cytoplasm.^35^ In our study, CASP2 was upregulated and then cleaved after *HOTAIR* knockdown, and siCasp2 rescued the apoptosis caused by *HOTAIR* knockdown, suggesting that CASP2 served as an initiator in this process. *Casp2* mRNA has been validated to be the target of miR-31,^36^ miR-34a,^37^ miR-17,^34^ and miR-96.^38^ In our study, we found *Casp2* was targeted by miR-125a-5p in HCT116, HeLa, HepG2, A549 and DU145 cells. MiR-125a-5p diminished the cleavage of CASP2 and inhibition of miR-125a-5p aggravated that, implying that *HOTAIR* indirectly modulates the translation and activation of *Casp2* via miR-125a-5p. In addition, we found that P53 was upregulated after *HOTAIR* knockdown, and confirmed that *P53* was a target of miR-125a-5p in colon cancer cells, which implied that P53 also had a role in the apoptotic pathway triggered by *HOTAIR* inhibition. In summary, when *HOTAIR* was knocked down, the level of miR-125a-5p therewith descended, and thus *Casp2* and *P53* mRNA was released. The increased CASP2 and P53 caused apoptosis.

Reduced expression of *Casp2* was reported in blood cancer and several solid tumors.^39, 40, 41^ High *Casp2* level is associated with poor survival in human neuroblastoma patients.^42^ Even so, mutations of *CASP2* are rare in various human cancers.^43, 44^ Therefore, direct mutational inactivation of *CASP2* might not fully explain its decline or loss-of-function in human tumors. In our study, we observed for the first time that *Casp2* was downregulated in clinical colon cancer tissues. The elevated expression of miR-125a-5p and its targeting effect on *Casp2* might indirectly explain *CASP2*' dereliction of duty in cancer.

In conclusion, we find targeting *HOTAIR* can induce cancer cell apoptosis, in a manner depending on reducing the level of miR-125a-5p and activating of apoptotic initiator CASP2 and P53. Also, we find miR-125a-5p is an oncogene in colon cancer, and identified *Casp2* is a new target of miR-125a-5p. For the first time, we explain the molecular mechanism of the apoptosis induced by *HOTAIR* knockdown and this multi-layer modulation might be valuable in cancer treatment strategy exploration.

## Materials and Methods

### Cell culture

HCT116, HeLa, A549, DU145 and HepG2 were purchased from ATCC (Manassas, VA, USA). These cancer cell lines were cultured in Dulbecco's modified Eagle's high glucose medium supplemented with 10% fetal bovine serum, 50 IU/ml penicillin, and 50 mg/ml streptomycin (Invitrogen, Carlsbad, CA, USA) and were maintained at 37 °C in a humidified incubator in the presence of 5% CO_2_.

### Transfection

SiCasp2, siEZH2, siLSD1, six different siRNAs targeting HOTAIR (siHOT1–6) and scrambled negative control siRNA (siNC) were synthesized in GenePharma (Shanghai, China). Their sequences are listed in Supplementary Table S1. MicroRNA angomirs and antagomirs were purchased from RiboBio (Guangzhou, China). RNAs were transfected into cancer cells using Lipofectamine iMAX (Invitrogen).

### RNA extraction and quantitative real-time PCR

Total RNA was extracted from tissues or cultured cells using TRIzol reagent (Invitrogen). Reverse transcription reactions were performed using the reverse transcription system kit (Aidlab, Beijing, China). Quantitative real-time PCR analysis was performed in triplicate with a CFX96 real-time system (Bio-Rad, Hercules, CA, USA) using GoTaq qPCR Master Mix (Promega, Madison, WI, USA). Relative mRNA and miRNA expression levels were normalized to *GAPDH* or *U6* snoRNA and relative expression fold change was calculated with the 2^−ΔΔCT^ method. Bulge-Loop™ miRNA qRT-PCR Primer Sets were purchased from RiboBio. Other genes' primer sequences are provided in Supplementary Table S2.

### Western blot analysis

Antibodies against CASP2, CASP3, CASP7, PARP, EZH2 and P53 were purchased from Cell Signaling Technology (Danvers, BSN, USA). Antibody against GAPDH was purchased from Santa Cruz (Dallas, TX, USA). Antibody against BCL-2 was purchased from Proteintech (Chicago, IL, USA). Antibody against LSD1 was purchased from Abcam (Cambridge, UK).

### Flow cytometric analysis of apoptosis

Cells were harvested at the indicated time points. After double staining with FITC-Annexin V and propidium iodide (Beyotime, Jiangsu, China), cells were analyzed with a flow cytometry (FCM, FACS Calibur, BD Biosciences, Franklin Lakes, NJ, USA) equipped with CellQuest software (BD Biosciences). Measurements were repeated independently three times.

### RNA sequencing and expression analysis

Total RNA from cells transfected with siHOT1 for 6, 24 and 48 h was extracted using RNeasy mini kit (Qiagen, Venlo, The Netherlands). The quality and integrity of the RNA samples were examined using the Agilent 2100 Bioanalyzer (Agilent, Santa Clara, CA, USA). After poly(A) selection, RNA was fragmented and then converted into cDNA sequencing library by using a TrueSeq DNA library preparation kit (Illumina, San Diego, CA, USA). The cDNA was end-repaired, adaptor ligated, PCR amplified and then sequenced on the Illumina Hiseq2000 platform with 100-bp pair-end sequencing strategy. In all, 4 GB of raw data were obtained for each sample. The SOAP software (http://soap.genomics.org.cn/, BGI, Guangzhou, China) was used to align the filtered reads to gencode.v19.long_noncoding_RNAs.gtf downloaded from ENCODE. Gene expression was calculated using the RPKM method (reads per kilobase transcriptome per million reads).

### Luciferase reporter assay

In all, 200 bp of the *Casp2* and *P53* 3′ UTR sequence containing the putative miR-125a-5p binding sites or the mutant miR-125a-5p binding sites were cloned into the psiCHECK™-2 vector to generate psiCHECK™-2-CASP2, psiCHECK™-2-CASP2-mut, psiCHECK™-2-P53, psiCHECK™-2-P53-mut plasmids. Cells grown in 24-well plates were transfected with 50 nM negative control miRNA mimics (nc miRNA mimics) or 50 nM miR-125a-5p mimics. Six hours later, plasmids were transfected. Luciferase activity was assayed 24 h after transfection, using a dual-luciferase reporter assay system (Promega) according to the manufacturer's instructions. All transfection experiments were performed in triplicate.

### Clinical samples analysis

Totally 80 pairs of colorectal tumor tissues and adjacent noncancerous tissues were obtained from patients who underwent surgery at the ChaoYang Hospital between 2013 and 2014. All specimens were immediately frozen in liquid nitrogen and stored at −80 °C until RNA extraction. Informed consent was obtained from all the patients and no patient received chemotherapy or radiotherapy before surgery. Clinicopathologic data are listed in Table 1.

### Statistical analysis

The Mann–Whitney test was used to estimate the significance of differences between miR-125a-5p expression of two different clinical groups. Other data were analyzed using Student's *t*-test. Data are shown as mean values S.E.M., and *P*-value <0.05 was considered statistically significant. All statistical analyses were performed using GraphPad Prism Software (La Jolla, CA, USA).

## Figures and Tables

**Table 1:** Relationship of miR-125a-5p expression with clinicopathologic factors in colon cancer

**Clinicopathologic factors**	***N* (%),** ***N*****=80**	**Relative miR-125a-5p expression**	***P*****-value**
*Gender*			*P*=0.992
Male	51 (64)	3.49 (0.37–13.91)	
Female	29 (36)	5.06 (0.14–29.43)	
*Age*			
≤66	40 (50)	4.27 (0.14–27.16)	*P*=0.981
>66	40 (50)	3.86 (0.53–29.43)	
*Tumor size*			*P*=0.023
≤5 cm	51 (64)	2.88 (0.14–14.16)	
>5 cm	29 (36)	6.14 (0.65–29.43)	
*Lymph node metastasis*			*P*=0.037
Negative	46 (58)	2.90 (0.14–14.16)	
Positive	34 (42)	5.63 (0.37–29.43)	
*Clinical stage*			*P*=0.025
I–II	46 (58)	2.87 (0.14–14.16)	
III –IV	34 (42)	5.67 (0.37–29.43)	

## Abbreviations

*HOTAIR*: homeobox transcript antisense RNA
lncRNA: long non-coding RNA
PRC2: polycomb repressive complex 2
EZH2: enhancer of zeste homolog 2
LSD1: lysine-specific demethylase 1A
Casp2: caspase 2
siHOT: siRNA targeting *HOTAIR*
siNC: scrambled siRNA
CASP3: caspase 3
CASP7: caspase 7
PARP: poly ADP-ribose polymerase
In-miR-125a-5p: miR-125a-5p inhibitors
BCL-2: B-cell lymphoma 2
CARD: caspase recruitment domain
CASP9: caspase 9
BID: BH3 interacting domain death agonist
