# Supplementary material for: MiR-125a-5p decreases after long non-coding RNA HOTAIR knockdown to promote cancer cell apoptosis by releasing caspase 2
Source: Cell Death Dis. 2016 Mar 10;7(3):e2137–. doi: 10.1038/cddis.2016.41 (PMC4823942; doi:10.1038/cddis.2016.41)
Supplement: Supplementary Figure Legends [file cddis201641x1.doc]

**Figure S1.** *HOTAIR* knockdown leads to cancer cell apoptosis. (a) 50 nM siHOT1 led to HCT116 cells apoptosis in a time dependent manner. (b) Different amount of siHOT1 led to HCT116 cells apoptosis after transfection of 48 hours. (c) FCM detected the apoptotic situation of HCT116 cells transfected with 50 nM siHOT1-6 or siNC for 48 hours.

**Figure S2.** *P53* is targeted by miR-125a-5p. (a) P53 was up-regulated by transfection of 50 nM siHOT1 in HCT116 cells, while BCL-2 level was unchanged. (b) Schematic outlines the predicted binding sites of miR-125a-5p on *P53*. (c) Luciferase reporter assay confirmed the target effect of miR-125a-5p on *P53*. (c) Western blot confirmed the target effect of miR-125a-5p on *P53*. Bars represent the mean ± S.E.M. from three independent experiments. ** P<0.01 by Student’s t test. All experiments were conducted in three biological repeats.
