# Supplementary figures and images for: MiR-125a-5p decreases after long non-coding RNA HOTAIR knockdown to promote cancer cell apoptosis by releasing caspase 2
Source: Cell Death Dis. 2016 Mar 10;7(3):e2137–. doi: 10.1038/cddis.2016.41 (PMC4823942; doi:10.1038/cddis.2016.41)

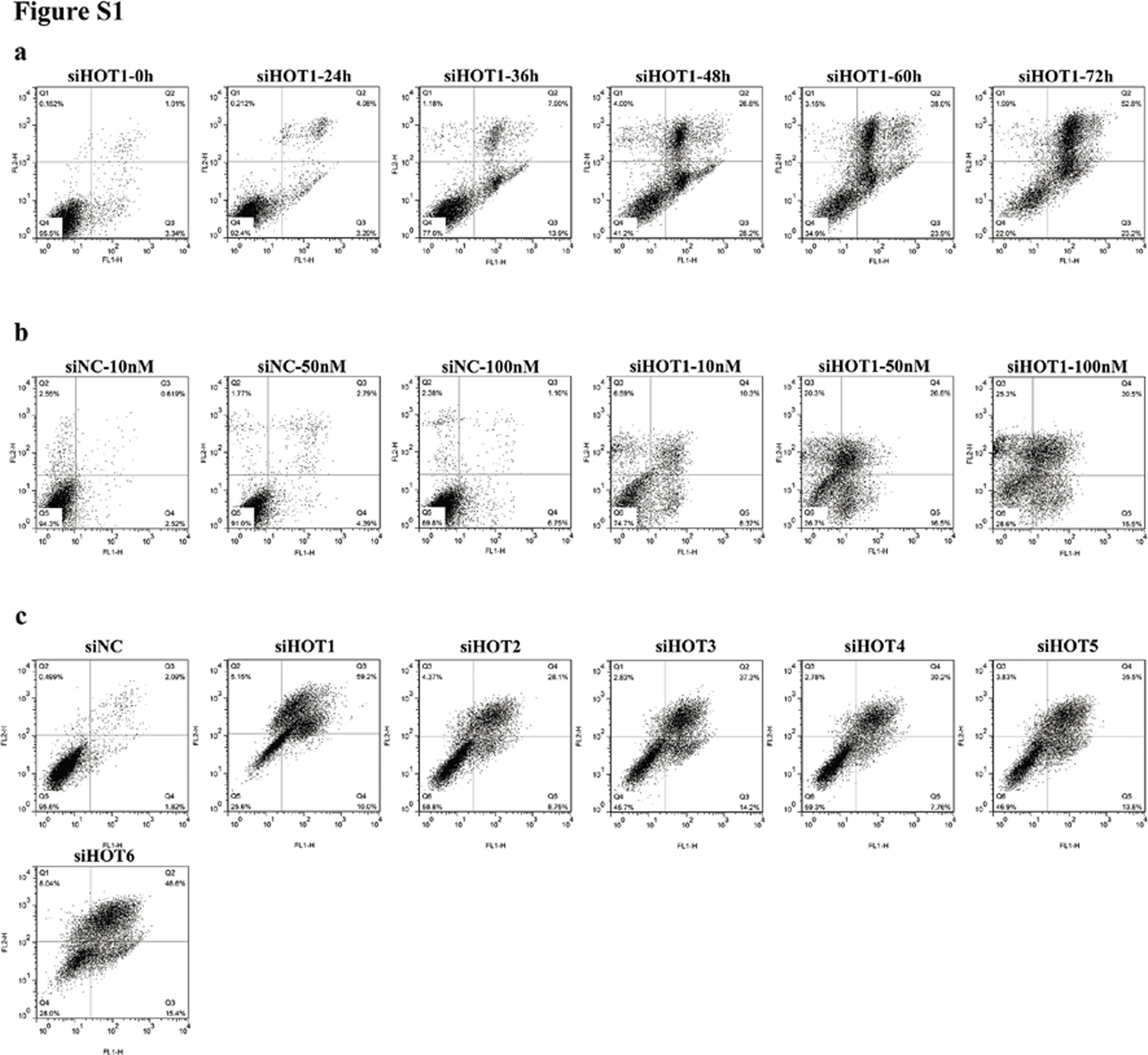

Supplement: Supplementary Figure S1 [file cddis201641x2.tif]

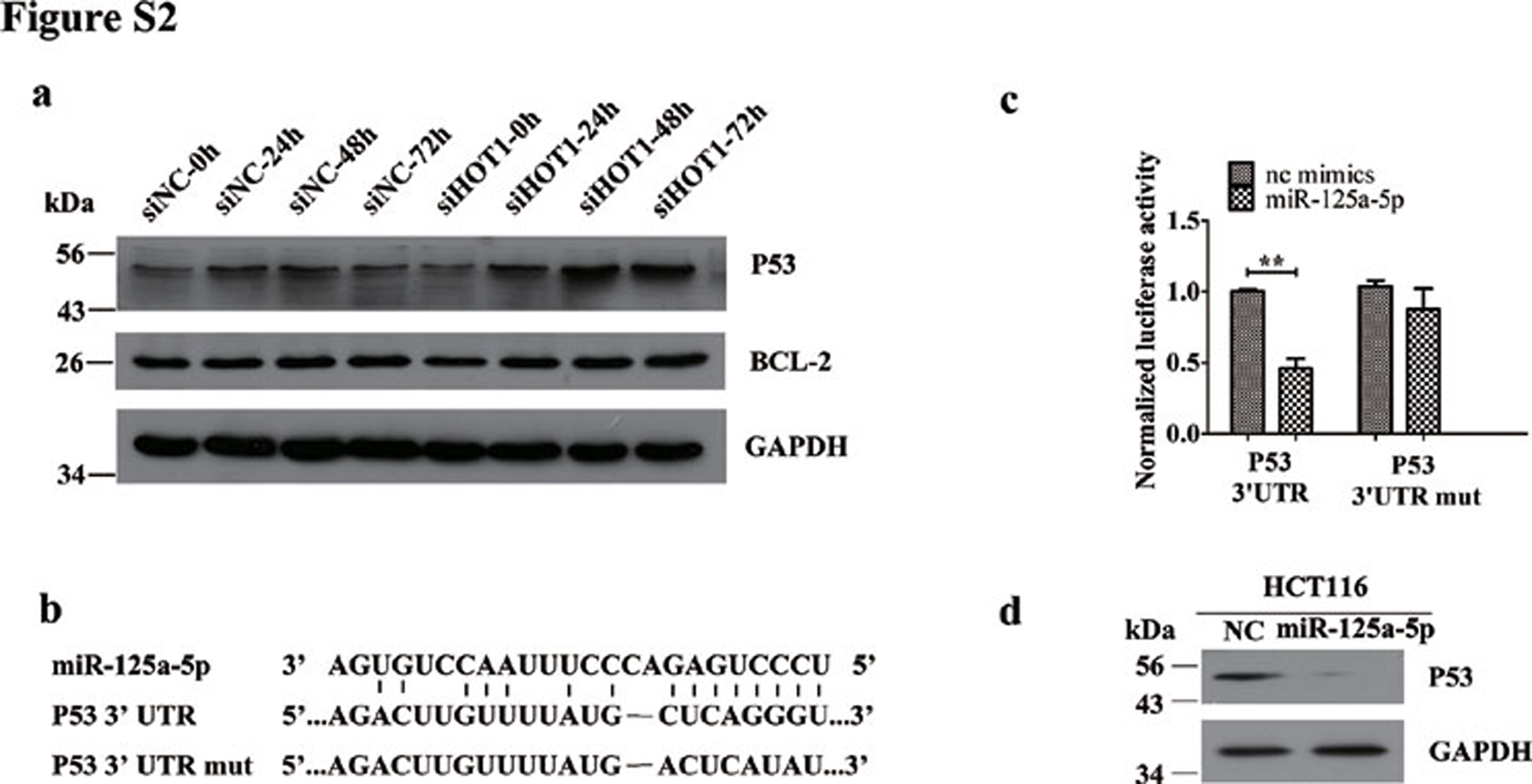

Supplement: Supplementary Figure S2 [file cddis201641x3.tif]
