# Supplementary material for: MiR-125a-5p decreases after long non-coding RNA HOTAIR knockdown to promote cancer cell apoptosis by releasing caspase 2
Source: Cell Death Dis. 2016 Mar 10;7(3):e2137–. doi: 10.1038/cddis.2016.41 (PMC4823942; doi:10.1038/cddis.2016.41)
Supplement: Supplementary Tables [file cddis201641x4.doc]

**Supplementary Data**

**Table S1**

| **siRNA** | **Sequence** |
| --- | --- |
| siHOTAIR1 | CCACAUGAACGCCCAGAGAUU |
| siHOTAIR2 | GAACGGGAGUACAGAGAGAUU |
| siHOTAIR3 | UAACAAGACCAGAGAGCUGUU |
| siHOTAIR4 | GAGGAAAAGGGAAAAUCUAUU |
| siHOTAIR5 | AAUUCUUAAAUUGGGCUGGUU |
| siHOTAIR6 | UUUUCUACCAGGUCGGUACUU |
| siCasp2 | ACAGCTGTTGTTGAGCGAAUU |
| siEZH2 | GAGGUUCAGACGAGCUGAUUU |
| siLSD1 | UGAAUUAGCUGAAACACAAUU |

**Table S2**

| **Gene names** |  | **Primer sequences** |
| --- | --- | --- |
| **HOTAIR** | Forward | ACATTCTGCCCTGATTTCCG |
|  | Reverse | CCGGTTTTTTCACCACATGTAA |
| **GAPDH** | Forward | AAGGTGAAGGTCGGAGTCAAC |
|  | Reverse | GGGGTCATTGATGGCAACAATA |
| **CASP2** | Forward | AGCTGTTGTTGAGCGAATTGT |
|  | Reverse | AGCAAGTTGAGGAGTTCCACA |
| **EZH2** | Forward | AATCAGAGTACATGCGACTGAGA |
|  | Reverse | GCTGTATCCTTCGCTGTTTCC |
| **LSD1** | Forward | TGACCGGATGACTTCTCAAGA |
|  | Reverse | GTTGGAGAGTAGCCTCAAATGTC |
